# Supplementary material for: Heterozygote advantage cannot explain MHC diversity, but MHC diversity can explain heterozygote advantage
Source: eLife. 2026 Jul 13;14:RP107256. doi: 10.7554/eLife.107256 (PMC13363215; doi:10.7554/eLife.107256)
Supplement: Peer review history

# Editor report: eLife Assessment

**DOI:** 10.7554/eLife.107256.3.sa0

**Authors:** Detlef Weigel

**Affiliations:** Max Planck Institute for Biology Tübingen Germany

**Keywords:** Solid, Valuable

This **valuable** study re-evaluates a published simulation model on the role of heterozygote advantage in shaping MHC diversity. By modifying key modeling assumptions, the author argues that the original conclusions depend on a narrow and potentially unrealistic parameter range. While the work is in principle **solid**, the robustness of this claim is viewed differently by the reviewers. The article further proposes an alternative modeling framework in which expansion of the MHC gene family allows homozygotes to outperform heterozygotes, thereby challenging the idea that heterozygote advantage alone can account for high allelic diversity at MHC loci. The topic is highly relevant for eco-immunology and evolutionary genetics, although it is not clear yet how well the model generalizes to other genes with different patterns of haplotype diversity in the population and different degrees of heterozygous advantage.

# Referee report: Reviewer #1 (Public review)

**DOI:** 10.7554/eLife.107256.3.sa1

The manuscript "Heterozygote advantage cannot explain MHC diversity, but MHC diversity can explain heterozygote advantage" explores two topics. First, it is claimed that the recently published by Mattias Siljestam and Claus Rueffler conclusion (in the following referred to as [SR] for brevity) that heterozygote advantage explains MHC diversity does not withstand an even very slight change in ecological parameters. Second, a modified model that allows an expansion of MHC gene family shows that homozygotes outperform heterozygotes. This is an important topic and could be of potential interest to the readership of eLife if the conclusions are valid and non-trivial.

The resubmitted manuscript addresses several questions from my previous review. In particular, there is a more detailed description of how the code of Siljestam and Rueffler ([SR]) was used for the simulations and the calculation of the factor 2.7 x 10^43 that is the key to the alleged breakdown of the numerical reasoning presented by in [SR].

Yet I think that important aspects of my critique of the first statement of the manuscript about the flaws of [SR] model remain unanswered. I guess the discussion becomes rather general about the universality and robustness of various types of models to parameter changes. My point is that none of the models is totally universal. The model in [SR] is not phenomenological as none of the parameters or functional forms were derived empirically. Instead, it is a proof of principle demonstration that inevitably grossly simplifies the actual immune response. The choice of constants and functions used in Eqs. (1-5) is dictated by the mathematical convenience and works in a limited range of parameter values. It is shown in [SR] that for 3 pathogens and reasonable "virulence " \nu, the alleles branch. These conclusions are supported by the analytically derived Adaptive Dynamics branching criteria (7), which, contrary to the statement is the cover letter (" It is clear from Fig. 4 of Siljestam and Rueffler that the branching condition is far from sufficient for high MHC diversity.") is perfectly confirmed by the simulation data shown in Fig. 4.

The mathematical simplicity of the [SR] model generates various artifacts, such as the mentioned by the Author reduction of the "condition" by an enormous factor 2.7 x 10^43 and the resulting decrease in the "survival" induced by the addition of a new pathogen. This occurs at the very large value of \nu=20, whose effect is enormous due to the Gaussian form of (1), which, once again, was chosen for the mathematical convenience. In reality, a new pathogen cannot reduce the "survival" by such a factor as it would wipe out any resident population. So to compensate for such an artifact, the additional factor c_max was introduced to buffer such an excess. There is no reason to fix c_max once for an arbitrary number of pathogens, because varying c_max basically reflects the observation that a well-adapted individual must have a reasonable survival probability. At the same time, there are many ways in which the numerical simulation may break down when the survival rates become of the order of 10^(-43) instead of one, so it comes to no surprise that the diversification, predicted by the adaptive dynamics, does not readily occur in the scenario with an addition or removal of the 8th pathogen with a very high virulence \nu=20.

I have doubts that the reported breakdown of the [SR] model with fixed c_max remains observable with less extreme values of m and \nu (say, for \nu=7 and m=3 plus or minus 1 used in Fig. 3 in the manuscript).

So I still find the claim that " the phenomenon that leads to high diversity in the simulations of Siljestam and Rueffler depends on finely tuned parameter values" is not well substantiated.

# Referee report: Reviewer #2 (Public review)

**DOI:** 10.7554/eLife.107256.3.sa2

Summary:

This study addresses the population genetic underpinnings of the extraordinary diversity of genes in the MHC, which is widespread among jawed vertebrates. This topic has been widely discussed and studied, and several hypotheses have been suggested to explain this diversity. One of them is based on the idea that heterozygote genotypes have an advantage over homozygotes. While this hypothesis lost early on support, a reason study claimed that there is good support for this idea. The current study highlights an important aspect that allows us to see results presented in the earlier published paper in a different light, changing strongly the conclusions of the earlier study, i.e., there is no support for a heterozygote advantage. This is a very important contribution to the field. Furthermore, this new study presents an alternative hypothesis to explain the maintenance of MHC diversity, which is based on the idea that gene duplications can create diversity without heterozygosity being important. This is an interesting idea, but not entirely new.

Strength:

(1) A careful re-evaluation of a published model, questioning a major assumption made by a previous study.

(2) A convincing reanalysis of a model that, in the light of the re-analysis-loses all support.

(3) A convincing suggestion for an alternative hypothesis.

Weakness:

(1) The title of the study is catchy, but it is explained only in the very end of the paper.

# Author comment: Author response

**DOI:** 10.7554/eLife.107256.3.sa3

**Authors:** Joshua L Cherry

**Affiliations:** National Institutes of Health Bethesda United States

The following is the authors’ response to the current reviews.

**Reviewer #1:**

Yet I think that important aspects of my critique of the first statement of the manuscript about the flaws of [SR] model remain unanswered.

I believe that I have fully addressed the points in the earlier review. The reviewer had doubted that my results were correct, attributing them to “a poor setup of the model” on my part. The reviewer stated that if I were correct about the factor of >10^43^ change in *c*max, this would “naturally break down all the estimates and conclusions made in Siljestam and Rueffler” (S&R).

It appears that the reviewer is now convinced that my results represent a faithful analysis of the models on which S&R based their claims. The reviewer now contends that these results, including the factor of >10^43^, present no difficulties for the claims of S&R after all. In fact, this enormous factor of >10^43^ is now claimed to *support* the conclusions of S&R by invalidating my conclusions. I respond to these new and very different arguments in what follows.

As I stated in the first round of review, the issue is not the enormity of this factor *per se*, but the fact that the compensatory adjustment of *c*max conceals the true effects of changes in other parameters. These effects are large; small changes to the parameter values mostly eliminate the diversity that the model is claimed to explain.

The model in [SR] is not phenomenological as none of the parameters or functional forms were derived empirically. Instead, it is a proof of principle demonstration that inevitably grossly simplifies the actual immune response.

The hidden sensitivity of the results of S&R to paramater values is sufficient to invalidate them as a proof of principle. The manuscript goes further and explains how the problem "is not specific to the details of the models of Siljestam and Rueffler, but is inherent in the phenomenon invoked to allow high diversity" because "any change that affects condition by as much as the difference between MHC heterozygotes and homozygotes will eliminate high equilibrium diversity". This general principle addresses all of the reviewer's points.

In reality, a new pathogen cannot reduce the "survival" by such a factor as it would wipe out any resident population. So to compensate for such an artifact, the additional factor *c*max was introduced to buffer such an excess. There is no reason to fix *c*max once for an arbitrary number of pathogens, because varying *c*max basically reflects the observation that a well-adapted individual must have a reasonable survival probability.

This is not a legitimate reason for making compensatory, diversity-promoting adjustments to *c*max when evaluating sensitivity to other parameters. If the number of pathogens or their virulence changes, *c*max obviously does not automatically change along with it. If the population or species consequently goes extinct, then it goes extinct. If it persists, it does so with the same value of *c*max.

The possibility of extinction arguably puts a minimum value on *c*max, but it does not restrict it to a range of values that conveniently leads to high MHC diversity. In the examples that I analyzed, slightly decreasing the number of pathogens or their virulence, which increases survivability, eliminates diversity. This phenomenon obviously cannot be dismissed on the grounds that survivability would be too low for the species to exist.

S&R in effect assume that the condition of the most fit homozygote remains fixed, regardless of the number of pathogens, their virulence, and myriad other differences between species. It is this assumption that is without justification.

At the same time, there are many ways in which the numerical simulation may break down when the survival rates become of the order of 10^(-43) instead of one

I am not sure what is meant by “the numerical simulation may break down”. Numerical error is not a tenable explanation of the lack of diversity observed in that simulation. The outcome is exactly what is expected from purely theoretical considerations: conditions of all genotypes fall on the steep part of the curve, making the mechanism proposed by S&R largely inoperative, so a pair of alleles forming a fit heterozygote comes to predominate. The numerical simulation is actually superfluous.

Low survival rates are completely irrelevant to the effect of *decreasing* the number of pathogens or their virulence, which does not lower survival rates, but does eliminate diversity.

so it comes to no surprise that the diversification, predicted by the adaptive dynamics, does not readily occur in the scenario with an addition or removal of the 8th pathogen with a very high virulence \nu=20.

Whether or not it surprising, the lack of diversity is a problem for the claims of S&R, as there is no reason to expect the number of pathogens to have just the right value to produce high diversity. Furthermore, for many combinations of values of the other parameters (e.g., my v=19.5 and 20.5 examples), no number of pathogens leads to high diversity.

Again, the general principle mentioned above makes the details that the reviewer refers to irrelevant. Nonetheless, some additional remarks are in order:

(1) This comment ignores the fact that removal of a pathogen, or a slight decrease in “virulence”, eliminates diversity without lowering survival rates.

(2) Small increases or decreases in *v* (virulence) eliminate diversity without having such large effects on condition.

(3) In the example emphasized by the reviewer, mean survival rates are nowhere near as low as 10^-43^. Only homozygotes have such low fitness.

(4) The adaptive dynamics predict the low diversity seen in the simulations, contrary to what the reviewer seems to suggest. Elimination of diversity is not an artifact of the simulation.

(5) *v*\=20 was chosen because it is most favorable to the model of S&R in that it yields the highest diversity. Indeed, S&R only observed realistically high diversity with the narrow gaussians that the reviewer objects to. With lower values of *v*, diversity is much lower, but even this meager diversity is eliminated by small changes in parameter values (see below). If narrow gaussians and large effects of pathogens somehow invalidate results, then they invalidate the high-diversity results of S&R.

I have doubts that the reported breakdown of the [SR] model with fixed *c*max remains observable with less extreme values of m and \nu (say, for \nu=7 and m=3 plus or minus 1 used in Fig. 3 in the manuscript).

These doubts are unwarrented. With the suggested parameter values, for example, increasing or decreasing *m* by 1 reduces the effective number of alleles to around 1 or 2. This can easily be checked using the simulation code of S&R, as detailed in my initial response and now in a Supplementary Text. Even without this result, the general principle mentioned above tells us that considering other regions of parameter space cannot rescue the conclusions of S&R.

So I still find the claim that " the phenomenon that leads to high diversity in the simulations of Siljestam and Rueffler depends on finely tuned parameter values" is not well substantiated.

What is unsubstantiated is the claim of S&R that “For a large part of the parameter space, more than 100 and up to over 200 alleles can emerge and coexist”. As my manuscript illustrates, this is an illusion created by the adjustment of one parameter to compensate for changes in others.

The reviewer even acknowledges that “the choice of constants and functions...works in a limited range of parameter values”. Furthermore, the manuscript explains why this problem is inherent to the general phenomenon, not specific to the details of the model or parameter values.

The following is the authors’ response to the original reviews.

**Public Reviews:**

**Reviewer #1 (Public review):**

It appears obvious that with no or a little fitness penalty, it becomes beneficial to have MHC-coding genes specific to each pathogen. A more thorough study that takes into account a realistic (most probably non-linear in gene number) fitness penalty, various numbers of pathogens that could grossly exceed the self-consistent fitness limit on the number of MHC genes, etc, could be more informative.

The reviewer seems to be referring to the cost of excessively high presentation breadth. Such a cost is irrelevant to the inferior fitness of a polymorphic population with heterozygote advantage compared to a monomorphic population with merely doubled gene copy number. It is relevant to the possibility of a fitness valley separating these two states, but this issue is addressed explicitly in the manuscript.

An addition or removal of one of the pathogens is reported to affect "the maximum condition", a key ecological characteristic of the model, by an enormous factor 10^43, naturally breaking down all the estimates and conclusions made in [RS]. This observation is not substantiated by any formulas, recipes for how to compute this number numerically, or other details, and is presented just as a self-standing number in the text.

It is encouraging that the reviewer agrees that this observation, if correct, would cast doubt on the conclusions of Siljestam and Rueffler. I would add that it is not the enormity of this factor *per se* that invalidates those conclusions, but the fact that the automatic compensatory adjustment of *c*max conceals the true effects of removing a pathogen, which are quite large.

I am not sure why the reviewer doubts that this observation is correct. The factor of 2.7∙10^43^ was determined in a straightforward manner in the course of simulating the symmetric Gaussian model of Siljestam and Rueffler with the specified parameter values. A simple way to determine this number is to have the simulation code print the value to which *c*max is set, or would be set, by the procedure of Siljestam and Rueffler for different parameter values. I have in this way confirmed this factor using the simulation code written and used by Siljestam and Rueffler. A procedure for doing so is described in the new Supplementary Text S1. In addition, I now give a theoretical derivation of this factor in Supplementary Text S2.

This begs the conclusion that the branching remains robust to changes in *c*max that span 4 decades as well.

That shows at most that the results are not extremely sensitive to *c*max or *K*. They are, nonetheless, exquisitely sensitive to *m* and *v.* This difference in sensitivities is the reason that a relatively small change to *m* leads to such a large compensatory change in *c*max. It is evident from Fig. 4 of Siljestam and Rueffler that the level of diversity is not robust to these very large changes in *c*max, which include, as noted above, a change of over 43 orders of magnitude.

As I wrote above, there is no explanation behind this number, so I can only guess that such a number is created by the removal or addition of a pathogen that is very far away from the other pathogens. Very far in this context means being separated in the x-space by a much greater distance than 1/\nu, the width of the pathogens' gaussians. Once again, I am not totally sure if this was the case, but if it were, some basic notions of how models are set up were broken. It appears very strange that nothing is said in the manuscript about the spatial distribution of the pathogens, which is crucial to their effects on the condition c.

I did not explicitly describe the distribution of pathogens in antigenic space because it is exactly the same as in Siljestam and Rueffler, Fig. 4: the vertices of a regular simplex, centered at the origin, with unity edge length.

The number in question (2.7∙10^43^) pertains to the Gaussian model with *v*\=20. As specified by Siljestam and Rueffler, each pathogen lies at a distance of 1 from every other pathogen, so the distance of any pathogen from the others is indeed much greater than 1/*v*. This condition holds, however, for most of the parameter space explored by Siljestam and Rueffler (their Fig. 4), and for all of the parameter space that seemingly supports their conclusions. Thus, if this condition indicates that “basic notions of how models are set up were broken”, they must have been broken by Siljestam and Rueffler.

...the branching condition appears to be pretty robust with respect to reasonable changes in parameters.

It is clear from Fig. 4 of Siljestam and Rueffler that the branching condition is far from sufficient for high MHC diversity.

Overall, I strongly suspect that an unfortunately poor setup of the model reported in the manuscript has led to the conclusions that dispute the much better-substantiated claims made in [SD].

The reviewer seems to be suggesting that my simulations are somehow flawed and my conclusions unreliable. I have addressed the reasons for this suggestion above. Furthermore, I have confirmed the main conclusion—the extreme sensitivity of the results of Siljestam and Rueffler to parameter values--using the code that they used for their simulations, indicating that my conclusions are not consequences of my having done a “poor setup of the model”. I now describe, in Supplementary Text S1, how anybody can verify my conclusions in this way.

**Reviewer #2 (Public review):**

(1) The statement that the model outcome of Siljestam and Rueffler is very sensitive to parameter values is, in this form, not correct. The sensitivity is only visible once a strong assumption by Siljestam and Rueffler is removed. This assumption is questionable, and it is well explained in the manuscript by J. Cherry why it should not be used. This may be seen as a subtle difference, but I think it is important to pin done the exact nature of the problem (see, for example, the abstract, where this is presented in a misleading way).

I appreciate the distinction, and the importance of clearly specifying the nature of the problem. However, as I understand it, Siljestam and Rueffler do not invoke the implausible assumption that changes to the number of pathogens or their virulence will be accompanied by compensatory changes to *c*max. Rather, they describe the adjustment of *c*max (Appendix 7) as a “helpful” standardization that applies “without loss of generality”. Indeed, my low-diversity results could be obtained, despite such adjustment, by combining the small change to *m* or *v* with a very large change to *K* (e.g., a factor of 2.7∙10^43^). In this sense there is no loss of generality, but the automatic adjustment of *c*max obscures the extreme sensitivity of the results to *m* and *v*.

(2) The title of the study is very catchy, but it needs to be explained better in the text.

I have expanded the end of the Discussion in the hope of clarifying the point expressed by the title.

**Recommendations for the authors:**

**Reviewer #1 (Recommendations for the authors):**

I would like to suggest to the author that they provide essential details about their simulations that would justify their claims, and to communicate with Mattias Siljestam and Claus Rueffler whether claims of the lack of robustness could be confirmed.

The models simulated were modified versions of those of Siljestam and Rueffler. Thus, only the modifications were described in my manuscript. I have added a more detailed description of how *c*max was set in the simulations concerned with sensitivity to parameter values. In addition, the new Supplementary Text S1, which describes confirmation of the lack of robustness using the code of Siljestam and Rueffler, should remove any doubt about this conclusion.

**Reviewer #2 (Recommendations for the authors):**

I have no further recommendations. The manuscript is well written and clear.

Thank you.

**Reviewer #3 (Recommendations for the authors):**

(1) Since this is a full report and not just a letter to the editor, it would benefit from a bit more introduction of what the MHC actually is and what the current understanding of its evolution is. Currently, it assumes a lot of knowledge about these genes that might not be available to every reader of eLife.

I have added some more information to the opening paragraph. I would also note that this report was submitted as a “Research Advance”, which may only need “minimal introductory material”.

(2) Some more recent literature on MHC evolution should be added, e.g., the review by Radwan et al. 2020 TiG, a concrete case of MHC heterozygote advantage by Arora et al. 2020 MolBiolEvol, and a simulation of MHC CNV evolution by Bentkowski et al. 2019 PLOSCompBiol.

I have cited some additional literature.

(3) Since much of the criticism hinges on the cmax parameter, its biological meaning or role (or the lack thereof) could be discussed more.

I am not sure what I can add to what is in the first paragraph of the Discussion.

(4) I find it difficult to grasp how the v parameter, which is intended to define pathogen virulence, if I understand it correctly, can be used to amend the breadth of peptide presentation. Maybe this could be illustrated better.

I have attempted to make this clearer. The parameter v actually controls the breadth of peptide detection conferred by an allele, which, if not identical to the breath of presentation, is certainly affected by it. The basis of the “virulence” interpretation seems to be that narrower detection breadth can, according to the model, only decrease peptide detection probability, which increases the damage done by pathogens.

(5) Please check sentences in lines 279ff on peptide detection and cost of . There seem to be words missing.

There was an extraneous word, which I have removed. Thank you for pointing this out.
